# Phage Therapy for Urinary Tract Infections: Progress and Challenges Ahead

**DOI:** 10.1007/s00192-025-06136-8

**Published:** 2025-05-13

**Authors:** Chase J. Morgan, Haley Atkins, Alan J. Wolfe, Linda Brubaker, Saima Aslam, Catherine Putonti, Michael B. Doud, Lindsey A. Burnett

**Affiliations:** 1 School of Biological Sciences, Division of Molecular Biology, University of California San Diego, La Jolla, CA 92093 USA; 2 Bioinformatics Program, Loyola University Chicago, Chicago, IL USA; 3 Department of Microbiology and Immunology, Loyola University Chicago, Maywood, IL USA; 4 Division of Urogynecology and Reconstructive Pelvic Surgery, Department of Obstetrics, Gynecology, and Reproductive Sciences, UC San Diego, 9300 Campus Point Dr, Mail Code 7433, La Jolla, CA 92037 USA; 5 Center for Innovative Phage Applications and Therapeutics, La Jolla, CA USA; 6 Division of Infectious Diseases and Global Public Health, Department of Medicine, University of California San Diego, 9500 Gilman Dr, Mail Code 0116, La Jolla, CA USA; 7 Department of Biology, Loyola University Chicago, Chicago, IL USA

**Keywords:** Phage therapy, Urinary phage, Urinary tract infection, Urinary virome

## Abstract

**Introduction and Hypothesis:**

Urinary tract infection (UTI) treatment is a growing public health concern owing to increasing antimicrobial resistance. Phage therapy, an alternative or adjunctive treatment to antibiotics, has the potential to address this challenge. However, clinical use of phage therapy is hindered by knowledge gaps and inconsistent reporting. The objective was to review the current state of phage therapy for UTIs and highlight research priorities that can optimize phage clinical efficacy.

**Methods:**

Current literature on UTI phage therapy was examined, focusing on the lack of standardized phage susceptibility testing, phage characterization, and microbiological assessments during and after treatment.

**Results:**

Critical areas requiring further investigation include appropriate phage dosing, optimal routes of administration, and the dynamics of phage–host and phage–patient interactions. The influence of the urinary microbiome, including endogenous phages, on treatment outcomes also needs to be better understood. Suggested data collection and reporting standards should be developed and implemented to improve clinical impact of studies examining phage therapy for UTI. Randomized clinical trials are needed to establish efficacy and determine the best practices for clinical use.

**Conclusion:**

Phage therapy is a promising alternative to antibiotics for managing UTIs, especially in the face of rising antimicrobial resistance. To fully realize its potential, however, future research must focus on standardized protocols, dosing strategies, and the role of the urinary microbiome, with an emphasis on rigorously conducted clinical trials. These steps are essential for integrating phage therapy into mainstream UTI treatment regimens.

## Introduction

Urinary tract infection (UTI) is one of the most common types of bacterial infection, affecting at least 150 million people annually worldwide based on epidemiological estimates over 2 decades ago [1], with recent estimates as high as 400 million [2]. Half of women will have at least one UTI during their lifetime and of these ~ 30% will develop recurrent UTI (defined as symptomatic culture-proven UTI at least twice in 6 months or three times in a year) [3]. Recurrent UTI is associated with the development of intracellular bacterial reservoirs, which may serve as the nidus for subsequent infections and reduce the efficacy of antibiotic therapy. Recurrent UTI is associated with repetitive and extended antibiotic use and poses significant therapeutic and public health issues owing to alarming increases in antibiotic resistance [4]. Additionally, the development of new antibiotics has been unable to keep up with the concerning rise in antibiotic resistance [5, 6], highlighting the need for novel therapeutic options. Current vaccine trials for UTI have had limited impact [7]. Here, we review the putative role of bacteriophage (phage) therapy as a therapeutic for UTI, highlight patient and microbiological factors that might impact successful treatment, and suggest reporting standards for future work to help to advance the field.

## Background on Phage Therapy

**Fig. 1:**
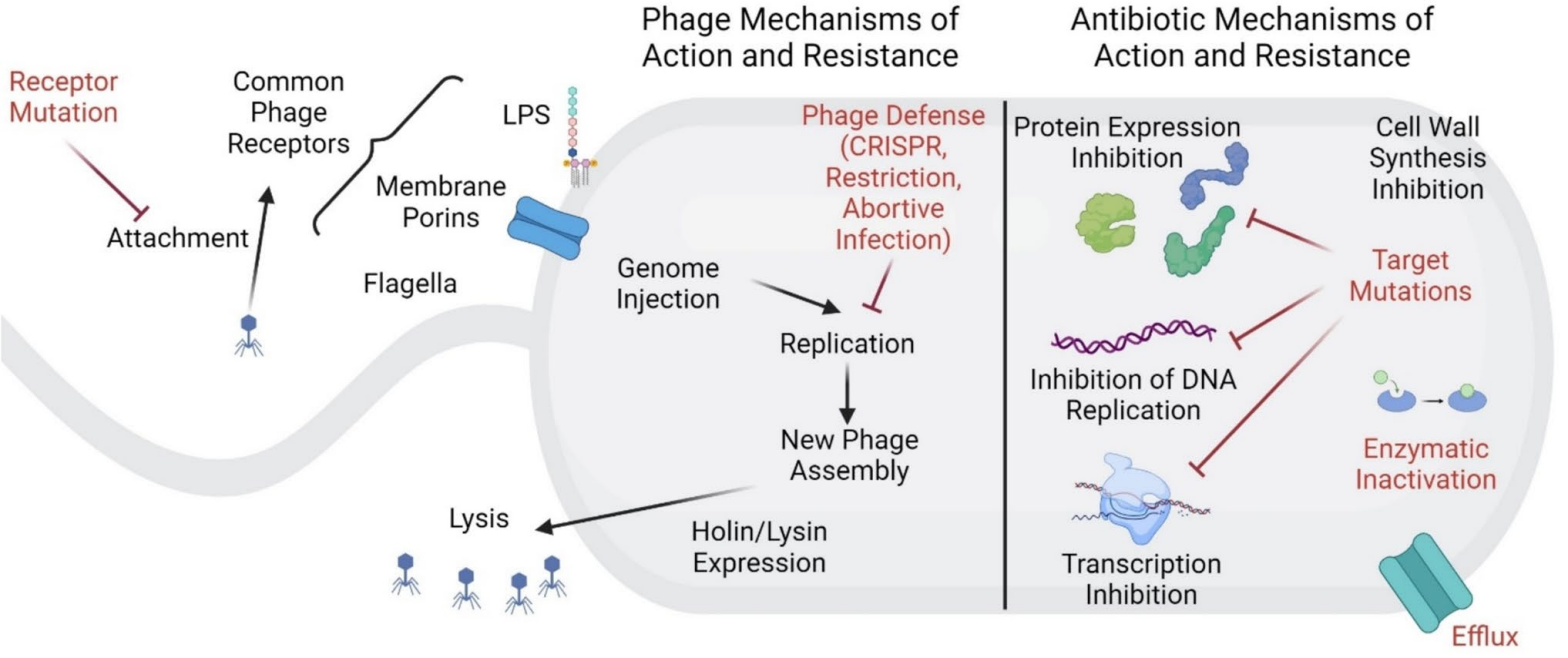
Overview of phage life cycle and bacterial interactions with phages and antibiotics. Antibiotic mechanisms of action (*black, right side*) include inhibition of cell wall synthesis, inhibition of protein expression, inhibition of DNA replication, and inhibition of transcription. To counteract these actions, bacteria antibiotic resistance mechanisms (*red, right side*) include targeted mutations, enzymatic inactivation, and efflux channels. Phage mechanisms of action against bacteria (*black, left side*) include attachment, genome injection, replication, assembly, holin/lysin expression, and ultimately lysis. Bacterial resistance mechanisms to phages include receptor mutations and phage defense mechanisms, including CRISPR, restriction of infection, and abortive infection. This figure was generated in Biorender

Phage therapy is the treatment of bacterial infections with naturally occurring bacterial viruses (phages), with or without genetic modification, that can selectively kill bacterial pathogens that cause infection [8]. These phages are isolated from the environment, replicated on causative bacterial species, purified, stringently tested for residual bacterial contamination, and then administered to the patient [9–16]. Although a variety of phages have been used for phage therapy, many therapeutic phages use a similar strategy to kill bacteria. These phages are typically lytic double-stranded DNA phages that bind to a specific receptor on the outside of the bacterial cell and inject their genome into the intracellular space of the bacterium (Fig. 1) [9–11]. Once inside, phage genes are expressed that hijack the physiology of the cell to manufacture more phage particles. Once these particles have assembled, the phage lyses the cell, killing the bacterium and releasing more phage particles that can target additional bacterial cells. This contrasts with the lysogenic lifestyle in which the phage genome becomes incorporated into the bacterial genome, a process considered undesirable in phage therapy as it is associated with bacterial acquisition of virulence factors. As such, phage therapy stands as a highly specific, and potentially self-amplifying, therapy to kill pathogens that cause a wide variety of bacterial infections.

Phage therapy was first explored concurrently or shortly after the discovery of phages. Frederic Twort and Felix D’Herrele are both credited with independently discovering phages in 1915 and 1917 respectively [17, 18]. The latter reported that phages isolated from patients with mild cases of dysentery could be used to treat other patients suffering from the illness [17]. Phage therapy continued to be explored for a variety of infections until the 1940s, when penicillin became widely available [17]. These early days of phage therapy had varying success, presumably owing to limits in the technology and biological understanding at the time. With the discovery of antibiotics, the Western medical establishment all but forgot phage therapy; some parts of the world, particularly in eastern Europe and the Caucasus, continued to explore medical phage applications [17–19]. For example, the Eliava Institute in Tbilisi, Georgia, has been supplying phages for therapeutic use for over 100 years [19]. However, only recently has phage therapy begun to be investigated with the level of scrutiny and evidence required for phages to become an FDA-approved intervention. The evidence for phage therapy is currently restricted to case studies, case series, and small clinical trials. Large-scale clinical trial data are still lacking.

The rise of widespread antimicrobial resistance has created a critical need for both novel therapies and an understanding of how these therapies interact with antibiotics [20]. Phage therapy has benefits both as an alternative to antibiotics and as an adjunctive therapy to antibiotics. Compared with antibiotics, phages have a very narrow spectrum, frequently infecting only a fraction of strains of a single species or a small number of related species of bacteria. In contrast, antibiotics can kill or inhibit large numbers of diverse bacterial species. This both harms the recipient’s beneficial microbiota and places selective pressure on the nonpathogenic microbiota to evolve antibiotic resistance. Antibiotic resistance genes in the resident microbiota can then be transferred to pathogens through horizontal gene transfer, increasing the risk of hard-to-treat infections [21]. Treatment with phages alone could bypass these side effects of antibiotics. However, treatment with phages and antibiotics could also be an effective strategy. Recent studies have elucidated key components of the interplay between phages and antibiotics. Several studies have demonstrated that phages that infect *Pseudomonas aeruginosa* can replicate in the presence of antibiotics and have discovered phages that prefer to replicate in and kill metabolically dormant persister cells, which antibiotics cannot kill [22–24]. Phages that can kill dormant bacteria may provide better outcomes in difficult-to-treat biofilm infections, which often involve dormant cells, although this has not yet been studied directly in the context of UTI. Some phages also encode biofilm matrix-degrading enzymes, which may increase the clinical effectiveness of antibiotics or phages [25, 26]. Furthermore, bacteria can become resistant to phages during the course of treatment, but knowledge of phage resistance mechanisms can allow us to use this to our advantage [27–29]. Phages have been isolated that bind outer membrane components of multi-drug efflux pumps that confer antibiotic resistance. Resistance to these phages occurs through mutation of this component of the multi-drug efflux pump. The mutations often lead to loss of function, which sensitizes the bacterium to antibiotics to which it was previously resistant, thus turning an antibiotic-resistant infection into an antibiotic-sensitive one [29]. Lipopolysaccharide (LPS) on the outer membrane of Gram-negative bacteria is another common phage receptor. Resistance to these LPS receptor phages occurs through loss of some LPS components, leading to decreased pathogenicity and increased antibiotic sensitivity [27]. Evolutionary trade-offs required for phage resistance and/or phage ability to clear dormant cells may lead to treatment benefits not seen with antibiotics alone. While best practices for administering phages alone or in combination with antibiotics has yet to be elucidated through clinical studies, these laboratory findings suggest ways to approach the problem in the clinic.

Studies have shown phage therapy to be generally safe [30]. Administration of phages in both compassionate use cases and in the context of clinical trials have not led to any reported severe adverse events (see Table 2) [13–16, 30, 41–43, 45–49]). Furthermore, phages for the purpose of food safety have received the “generally recognized as safe” designation from the FDA [50]. However, proper preparation, testing, and selection of phages is critical for maintaining the safety of phage therapy. The phages used for phage therapy must be lytic, without the ability to become lysogenic and with a low probability of transducing genes from one bacterium to another. Some lysogenic phages are known to carry toxins (e.g., Shiga toxin) and antibiotic resistance genes, and therefore are unfit for therapeutic use. In certain cases, lysogenic phages can be mutated to become obligately lytic and can be used in therapy [47], but this must be done carefully to avoid the risks of lysogeny.

## Endogenous Phages in Urine

Phages are naturally occurring and present in the urine virome, interacting with the bacterial microbiome in ways that may influence UTI progression and the effectiveness of phage therapy. Endogenous phages can alter both the physiology of bacteria in the urinary tract and their susceptibility to phage through superinfection exclusion, phage-encoded defense systems, and other mechanisms of phage–phage conflict [51–54]. Recent studies have found a diverse array of phages in urine samples from both healthy individuals and those with lower urinary tract symptoms (Table 1). Shotgun metagenomic sequencing of urine samples has identified phages as the most abundant viruses [31, 55]. Although these studies examined both bacterial and viral members of the urinary community, the predominance of phages has also been noted when only the viral constituents were sequenced. In the first study of the urinary virome by Santiago-Rodriguez et al., a well-balanced viral community within the urinary tract was detected, even if an individual had a UTI [33]. Most notably, it was found that > 99% of the viruses were phages. Similarly, a study of 40 healthy children found that as age increases, the relative abundance of tailed phages (*Caudoviricetes*) also increases, with *Shigella* phage SHFML- 11 being the most abundant [39]. However, there are also studies that do not find a robust community of phages in urine. For instance, a study of post-menopausal women with recurrent UTIs observed that the most common viral taxon was JC polyomavirus, not phages [32]. Similarly, a longitudinal study of kidney transplant recipients found that eukaryotic viruses were the most prominent members of the urinary virome, not phages [56]. Given just this handful of studies conducted to date, we can already speculate that the composition of the urinary virome might vary between participant populations.

**Table 1:** Endogenous phages infectious of urinary microbiome bacteria of interest

Host species	Identified from sequencing urinary virome^a^	Identified from sequencing urinary metagenome^a^	Isolated virions
Enterobacteriaceae		Moustafa et al. [31]; Neugent et al. [32]	
*Escherichia*	Santiago-Rodriguez et al. [33]		Dallas & Kingsbery [34]; Brown-Jaque et al. [35]; Malki et al. [36]; Rahimzadeh et al. [37]; Salih Doğan et al. [38]
*Klebsiella*			Salih Doğan et al. [38]
*Shigella*	Wehedy et al. [39]		
*Enterococcus*	Santiago-Rodriguez et al. [33]	Moustafa et al. [31]	
*Pseudomonas*		Moustafa et al. [31]	Brown-Jaque et al. [35]
*Lactobacillus*		Moustafa et al. [31]; Garretto et al. [40]	
*Streptococcus*		Moustafa et al. [31]; Garretto et al. [40]	

^a^Identification by sequencing relies on sequence homology to characterized phage. Thus, host prediction is tenuous

Temperate phages within the urinary microbiota likely play an important role in shaping the bacterial community, akin to what occurs within the gastrointestinal tract [57]. Induction, or the transition from the lysogenic life cycle to the lytic life cycle, has also been associated with symptoms of the GI tract, e.g., inflammatory bowel disease [58]. Identifying the factors that induce lytic transition is critical for understanding the dynamics of phage and bacteria within the human body [59]. Although phage–bacteria interactions have yet to be explored within the urinary microbiota, well-known external factors that can cause induction are particularly relevant for the female urinary microbiota, in particular pH and H_2_O_2_. Both are products of *Lactobacillus* species, which often dominate the healthy female urogenital microbiota [60–62]. Both pH and H_2_O_2_ have been used in the laboratory to induce phages from *E. coli* strains isolated from the bladder [63]. This suggests that increases in lactobacilli and other lactic acid bacteria in the urinary microbiota might increase temperate virion populations. Overall, additional research is needed to better understand how endogenous phages influence lower urinary tract symptoms and composition of urinary microbiota, as well as how phage therapy may interact with or affect these endogenous phages.

## Early Evidence Supporting Phage Therapy for Urinary Tract Infection

Urinary tract infections are an attractive target for phage therapy. UTIs that are frequently chronic or recurrent are caused by several pathogens of concern (e.g., *E. coli*, *K. pneumoniae*), and standard-of-care treatment relies on repeated administration of antibiotics associated with increasing the frequency of antibiotic resistance [1, 3–6]. UTIs can be treated through multiple modes of administration of phages including intravenous and intravesicular dosing. Although large-scale randomized controlled trial (RCT) data are still lacking, a number of case studies and a handful of small clinical trials have been performed evaluating the safety and efficacy of phage therapy for UTI (Table 2).

**Table 2:** Urinary tract infection phage therapy cases and clinical trials

Study	Study type	Target organism	Dose and administration	Concurrent antibiotics	Outcome
Leitner et al. [41]	Clinical trial	Various	PYO phage 20 ml (10^5^ units per ml) intravesically twice daily for 7 days	No	Bacterial clearance: non-inferior to antibiotics or placebo. CFU reduction: inferior to antibiotics at 7-day follow-up
Le et al. [42]	Case study	*K. pneumoniae*	Three-phage cocktail (5 x 10^9^ total PFU) intravenously twice daily for 4 weeks while the patient was asymptomatic	No	One recurrence with *K. pneumoniae* at 206 days post-phage, successfully treated with oral antibiotics
Gainey et al. [43]	Case study	ESBL *E. coli*	Two-phage cocktail (9 × 10^9^ PFU, 1 × 10^9^ PFU) intravenously daily for 3 weeks	Ertapenem (first 3 days only)	No ESBL *E. coli* for the 4 years of follow-up
Terwilliger et al. [44]	Case study	ESBL *E. coli*	Four-phage cocktail (1 × 10^9^ total PFU) intravenously twice daily for 2 weeks	Ertapenem	Two cases of asymptomatic bacteriuria not requiring antibiotics in 12-week follow-up
Kuipers et al. [45]	Case study	ESBL *K. pneumoniae*	Dose and number of phages unknown. Oral and intravesical, patient self-administered for 8 weeks	Meropenem	*K. pneumoniae* negative urine for 14 months
Kim et al. [46]	Clinical trial (ongoing)	*E. coli*	Single phage. Intravesically and intravenously at varying doses	Trimethoprim-sulfamethoxazole	16 patients treated with resolution of UTI by day 10. (phase 2, part 1, uncontrolled)

*ESBL* extended-spectrum beta-lactamase

A recent systematic review of phage therapy for UTI found 55 articles published in the last 100 years detailing results from human or animal studies [48]. Of note, four of these studies were reported to be RCTs in humans, but only one was published in a peer-reviewed journal, whereas the rest were conference abstracts [41, 48]. One peer-reviewed RCT was a 2021 double-blind, placebo-controlled study investigating the use of intravesical phage therapy for UTI in patients undergoing transurethral resection of the prostate [41]. It found that phages were non-inferior to both antibiotic and placebo at normalizing urine culture (< 10^4^ CFU/ml), but performed worse than antibiotics when comparing total CFU reduction post-treatment. This study randomized 113 patients into three arms: systemic antibiotics, intravesical phage instillation with a commercially available multi-species phage cocktail (20 ml of 10^4^–10^5^ PFU/ml), or intravesical placebo, with an approximately equal number of patients in each group. It is difficult to interpret the results of this trial though, as the causative organism varies between groups and the phage sensitivities of those organisms are not reported. Moreover, the phage cocktail was reported to be “adapted” periodically, but no information on this adaptation is provided and it is unclear which or if all patients received “adapted” phage therapy. Maybe the most significant result of this trial is that the number of adverse events was lowest in the phage group compared with the antibiotics and placebo groups and that no severe adverse events were observed.

A number of case studies show more promising results (Table 2) [42–45]. One 2022 case report discusses a 17-year-old renal transplant patient who was treated for recurrent extended-spectrum beta-lactamase (ESBL) *E. coli* urosepsis with a cocktail of two genetically distinct *E. coli* phages (EcAP29Phi234 and EcAP29Phi237, 10^9^ PFU each intravenously daily for 21 days) administered to treat a fourth recurrence of the infection after failed treatments with ertapenem and ceftazidime-avibactam [43]. After phage therapy, the patient cleared the infection, and during the 4 years of follow-up, the patient has not tested positive for ESBL *E. coli* again. Another case study from the Netherlands published in 2019 details a 58-year-old renal transplant patient with relapsing *K. pneumoniae* UTI, which he was unable to clear despite in vitro carbapenem sensitivity [45]. After seven failed courses of meropenem, this patient self-administered intravesical *K. pneumoniae* phages acquired from the Eliava Institute via intermittent catheterization, as well consuming the phages orally, for 4 weeks. Although phage susceptibility was not performed in advance, lysis was observed via plaque assay on some of his previous *K. pneumoniae* isolates, although the exact titer of the phage administered was not reported. This patient showed clinical and microbiological improvement following phage therapy, clearing the infection without microbiological recurrence after 14 months of follow-up. A third case study published in 2021 describes a 56-year-old male patient with a liver transplant who received phage therapy for recurrent ESBL *E. coli* prostate and UTI [44]. The patient was treated intravenously for 2 weeks with a cocktail of four *E. coli* phages at a final concentration of 10^9^ PFU/ml along with 6 weeks of intravenous ertapenem. Although the patient did not achieve full microbiological clearance, his symptoms improved to the point of asymptomatic bacteriuria and subsequent cultures revealed mutations in the bacterium that appear to have reduced the virulence.

When evaluating phage therapy case reports, for UTI or other conditions, it is important to note that these are done under compassionate use authorization when other treatment modalities fail. As such, these patients frequently have progressed infections and are currently receiving other treatments, making it difficult to ascertain the exact reason for treatment failure or success. RCTs are the gold standard for evidence-based medicine and well-reasoned and well-powered studies are still needed to evaluate the efficacy of phage therapy.

Five current or future UTI phage therapy clinical trials are listed on clinicaltrials.gov. The first is the ELIMINATE trial (NCT05488340), a double-blind phase 2 superiority study being conducted at multiple sites around the USA looking at the efficacy of an intravesical and intravenous recombinant phage cocktail given along with oral antibiotics for treating acute, uncomplicated *E. coli* UTI [46]. A phase 1 safety study of this phage was completed in 2022. The second is an active, single-patient, open-label phase I/II trial (NCT05537519) based in Canada looking at the safety and efficacy of a three-phage cocktail administered orally, topically, and intravesically to treat drug-resistant recurrent UTI. The third is a placebo-controlled phase 1/2 trial (NCT06409819) that will administer intravenous phage to asymptomatic female kidney transplant patients with recurrent *E. coli* or *K. pneumoniae* UTI. The fourth is an open label trial for intravesical bacteriophage treatment of recurrent ESBL Enterobacteriaceae UTIs in kidney transplant patients based in Iran (NCT05967130). The last is a phase 1 study (NCT06559618) investigating intravesical phage therapy for bacteriuria in patients with spinal cord injury. The results of these trials may provide a clearer picture of what administration methods (intravenous versus intravesical versus combination) are superior and what organisms or patient populations respond best to phage treatment.

## Immune Responses to Phage Therapy

In the course of phage therapy, there is the possibility for complex interactions with components of the innate and adaptive immune system that might impact the efficacy of phage treatment, either for better [64] or for worse [65–67]. Many factors can theoretically contribute to whether human immune responses affect efficacy, including the immunocompetency of the patient, the site of infection (e.g., in immunologically privileged compartments or with differences in tissue-resident immune cell responses), the route of administration (e.g., oral, intravenous, intravesicular, inhalation, percutaneous lavage, or cutaneous administration), the phage dose and frequency of administration, and phage-specific idiosyncrasies in the potential to stimulate the immune system.

Although phages cannot directly infect mammalian cells, they can undergo transcytosis [68] and endocytosis [65, 69]. Phage components can be recognized by the innate immune system as pathogen-associated molecular patterns that can modulate cytokine signaling and immune cell behavior [65, 70, 71]. These interactions with the innate immune system may be either beneficial [64] or detrimental [65], depending on the context. Similarly, variable results have been observed with respect to both the strength and the implications of humoral responses to phages. Neutralizing antibody activity appears to be related to the route of administration, and intravesicular administration for treatment of UTI may elicit lower levels of neutralizing antibodies compared with intravenous administration [72]. Furthermore, some phages in a cocktail may be more immunogenic than others [42, 66]. Neutralizing anti-phage antibodies are not universally observed [73, 74], and the presence of these antibodies may not always correlate with treatment failure [44, 49, 75], especially if the time lag associated with antibody development is longer than the treatment period required to clear the infection. When humoral responses do occur, detailed experiments are required to map the phage proteins targeted by antibodies and to what extent those specific antibodies impair phage predation of the target bacteria [66, 67]. More work is needed to understand the immune response to phage administration and how this may vary based on route, dose, and timing of therapy and the composition of the phages or phage cocktail administered.

## Considerations for Clinical Use of Phages

A significant consideration in phage therapy is the highly specific nature of phage–bacteria interactions. In current clinical practice, a patient bacterial isolate is commonly matched with a suitable therapeutic phage by screening existing phage collections. Even among highly related phages and isolates of a species of bacteria, there can be myriad phage–bacteria interactions [76, 77]. The bacterial and phage determinants of productive phage infection in clinical applications of phage therapy are insufficiently understood, and therapeutic phage selection must be performed empirically. Two main conceptual categories of interactions can be defined: those that occur extracellularly (i.e., phage recognition of receptor proteins leading to injection of genome into the host cell) and those that occur intracellularly (i.e., phage hijacking of host-cell machinery and host-cell defense systems that can recognize and respond to phage invasion).

To address this, pioneering work using genome-wide approaches has laid the groundwork for high-throughput delineation of phage receptors in laboratory strains of bacteria [78, 79]. However, there is still a paucity of literature surveying the impact of receptor variation across clinical isolates on phage infectivity. Future work is needed in the study of genetic and phenotypic variation in the expression of phage receptors in clinical isolates of bacteria. Receptor-sequence variation in these isolates, either point mutations or gene deletions, can complicate the generalization of knowledge on phage-receptor utilization from model strains in the research laboratory to pathogenic strains in the clinic. Furthermore, the in vivo infection milieu is a dynamic and complex environment where changes in pH, nutrient availability, and other biochemical factors can impact bacterial physiology in ways that modulate phage susceptibility by regulating expression of bacterial proteins that commonly serve as phage receptors [80–82]. Utilizing well-characterized phages or phage cocktails with known receptors and receptor binding sites can help us to predict how the host bacterium will respond to the phages. However, given the narrow host range of most phages, personalized use of less characterized phages that have been tested against the patient’s individual strain may prove to be more effective.

The intracellular interactions between phage and bacteria are intricate and highly specific to the phage and bacteria in question. There has been a recent explosion in the identification and understanding of an incredibly diverse repertoire of phage defense systems [83–85], and additional defense systems likely remain to be discovered. These defense systems are distributed heterogeneously throughout otherwise closely related isolates of the same bacterial species [83], and so caution is warranted when generalizing studies of phage–bacteria interactions in “model strains” of pathogenic bacteria. The mechanism of action of many of these systems remains enigmatic, and a flurry of ongoing work is characterizing how these diverse defense systems function and how phages can subvert them [86–93]. More research is needed in the UTI space to understand the role of endogenous phages, immune responses to phage therapy, the role of phage defense systems in clinical uropathogen isolates, and methods of modifying phage therapeutics to exhibit appropriate target range and optimize dosing, timing, and route of administration for efficacy.

It is still uncertain whether it is superior to give multiple phages as a cocktail or a single phage at a time. Both approaches have logical benefits and drawbacks. Cocktails can be made to cover a broader range of hosts, therefore allowing a more standardized treatment to be given to patients, despite differing causative strains. Cocktails may also slow or prevent the infection from evolving resistance to treatment, as phages can be selected that utilize different receptors. However, evidence suggests that, at least in laboratory settings, one phage frequently dominates and outcompetes the others. Thus, one phage is clearing the infection, whereas the other phages are merely passengers. These passenger phages, though, are being exposed to the immune system, which may cause the body to elicit an immune response against them and compromise the ability to use them in the future if the dominant phage fails. Furthermore, various cell types in the human body clear phages from circulation fairly rapidly, meaning that nondominant phages are unlikely to stay around long enough between treatments to become effective if resistance to the dominant phage develops. Treating with a single phage narrows the potential host range, with the benefit of a more controlled treatment. If the phage fails, we can treat subsequently with a different phage. If the therapy is initially effective, but the bacterium becomes resistant, we can better predict the phenotypic change that results from that resistance and adjust future treatments accordingly. Given the benefits and drawbacks of both approaches, we will likely see both single phage and phage cocktail treatments administered as needed based on clinical course, patient population, and clinical microbiology infrastructure.

## Suggested Data Collection and Reporting Standards when Treating Urinary Tract Infection with Phages

Although there are numerous reports of positive outcomes for UTI patients after phage therapy [48], critical evaluation of these reports is challenging. There is a collective push in the field to standardize the approach to phage therapy; however, no phage susceptibility testing or reporting standards currently exist, nor are there standards for follow-up microbiological testing or metrics for clinical improvement [94]. The high variability in how these cases and trials are both performed and reported makes it difficult or impossible to determine the reasons for success or failure. Personalized phage therapy shows immense promise as an adjunctive treatment for UTI, but it is critical that we establish testing, treatment, and reporting standards so that we can create evidence-based phage treatments for patients in need.

### Phage Susceptibility Testing

Phage susceptibility testing methods are not standardized. Both solid and liquid media assays are used, and there are many variables, including culture media, cell density, multiplicity of infection, and timing that can vary between protocols. Until standardized methods emerge, it is critical that all published studies clearly describe specific and reproducible protocols and the resulting data for the phages used against the organisms targeted for treatment. When cocktails of phages are used, it is important to include characterization of the individual phage components in addition to the cocktail, as phage–phage interactions within cocktails may be unpredictable. Robust phage susceptibility assays should also be used to assess phage stability over time in the storage conditions that treatment phages experience prior to patient administration.

### Genome Sequences of Phages and Bacteria

Phages and bacteria, even closely related strains, are genetically diverse, and understanding how this genetic heterogeneity impacts the success of phage therapy will be important in future research. Whole-genome sequencing has become inexpensive enough that it should be a standard element of data collection and reporting in published studies. High-quality bacterial genomes can be sequenced and assembled for ~$100 and sequencing of phage genomes can be even less expensive owing to the smaller genome sizes. Genome sequencing would clarify when similar or identical phages might have been otherwise inadvertently used in cocktails [95], can help researchers to identify genetic changes in phage or bacteria that may emerge during treatment, and will assist in identifying phage and bacterial genetic determinants of successful (or failed) therapy.

### Microbiological Monitoring During and After Phage Treatment

Phage therapy to treat UTI represents a unique opportunity for non-invasive sampling that is often more difficult at other infection sites. Prospectively planned urine collection for microbiological analysis permits quantitative measurements of phage and bacterial abundances that could provide insight into the dynamics of phage and bacterial population sizes during treatment in vivo in the urinary environment, which may differ considerably from laboratory conditions. Isolation of bacteria and phages during and after the course of treatment is important for characterizing phenotypic (especially phage and antibiotic susceptibility) and genomic changes in both bacteria and phages.

## Conclusion

Widespread and increasing antimicrobial resistance threatens effective clinical treatment of UTIs. Phage therapy is a promising investigative therapy for patients suffering from UTIs, both as an adjunctive therapy along with standard-of-care antibiotics, and as a replacement for antibiotics in appropriate patients. In order to maximize research contributions, phage therapy reports should adopt rigorous reporting standards for a variety of parameters. Enhanced reporting standardization would help to move the field forward efficiently and improve understanding of phage therapy efficacy in different contexts. Consensus regarding standardized phage susceptibility testing, the characteristics of phages used, and microbiological testing during and after treatment should be prioritized. Future research, especially in the form of randomized clinical trials, is needed to determine the most effective approach for using phages in the treatment of UTIs. Key areas of future research include phage dosing, phage administration route, phage–host interactions, phage–patient interactions, and how the urinary microbiome (including endogenous phages of the urinary virome) influences the effectiveness of phage treatment.
